# LOX-1 and Its Splice Variants: A New Challenge for Atherosclerosis and Cancer-Targeted Therapies

**DOI:** 10.3390/ijms18020290

**Published:** 2017-01-29

**Authors:** Barbara Rizzacasa, Elena Morini, Sabina Pucci, Michela Murdocca, Giuseppe Novelli, Francesca Amati

**Affiliations:** Department of Biomedicine and Prevention, University of Rome Tor Vergata, Via Montpellier 1, 00133 Rome; Italy; barbara.rizzacasa@gmail.com (B.R.); morinielena.em@gmail.com (E.M.); sabina.pucci@uniroma2.it (S.P.); miky.murdi@hotmail.it (M.M.); novelli@med.uniroma2.it (G.N.)

**Keywords:** *OLR1*, alternative splicing, atherosclerosis, cancer

## Abstract

Alternative splicing (AS) is a process in which precursor messenger RNA (pre-mRNA) splicing sites are differentially selected to diversify the protein isoform population. Changes in AS patterns have an essential role in normal development, differentiation and response to physiological stimuli. It is documented that AS can generate both “risk” and “protective” splice variants that can contribute to the pathogenesis of several diseases including atherosclerosis. The main endothelial receptor for oxidized low-density lipoprotein (ox-LDLs) is LOX-1 receptor protein encoded by the *OLR1* gene. When *OLR1* undergoes AS events, it generates three variants: *OLR1*, *OLR1D4* and *LOXIN.* The latter lacks exon 5 and two-thirds of the functional domain. Literature data demonstrate a protective role of LOXIN in pathologies correlated with LOX-1 overexpression such as atherosclerosis and tumors. In this review, we summarize recent developments in understanding of *OLR1* AS while also highlighting data warranting further investigation of this process as a novel therapeutic target.

## 1. Introduction

The accurate regulation of gene expression is fundamental to ensure the presence of the correct complement of RNAs and proteins in the correct cell at the right time. Due to its diversity, in sequence and structure, RNA plays crucial roles in cell biology [1]. The splicing of precursor mRNA (pre-mRNA) is a pivotal regulatory stage in gene expression that ensures the regulation of tissue specificity and quantity of mRNAs, and its dysregulation can lead to the development of many diseases [2,3,4]. The challenges for the splicing machinery (spliceosome) are to recognize exons, ignore pre-mRNA segments that resemble exons (pseudo-exons), join contiguous exons without inadvertent skipping and appropriately undergo alternative splicing to meet the physiological requirements of cells and tissues [3,5,6]. Alternative splicing (AS) is defined as the process by which splicing sites in pre-mRNAs are differentially selected and paired to produce multiple mature mRNAs and protein isoforms with specific structural and functional properties. Affecting approximately 95% of human gene expression, AS is a predominant source of human proteomic diversity.

## 2. Pre-mRNA Splicing Mechanism

**Figure 1:**
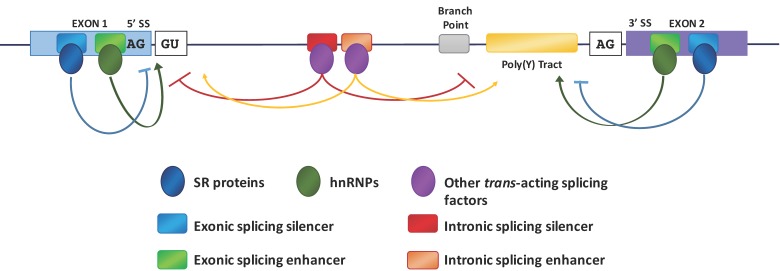
Model of mRNA splicing. The figure shows the key elements that regulate splicing: consensus sequences of the 5′ and 3′ splice sites, sequence elements required for assembly of the spliceosome onto the pre-mRNA such as the splice sites themselves, the polypyrimidine tract (poly(Y) tract), the branch point and enhancer and silencer elements that are binding regions for *trans*-acting splicing factors (serine/arginine-rich (SR) proteins, heterogeneous nuclear ribonucleoproteins (hnRNPs) and other proteins). Arrows, originating from exonic (ESE) or intronic splicing enhancers (ISE), indicate that the choice of a particular splice site is promoted; T-bars, originating from exonic (ESS) or intronic splicing silencers (ISS), indicate that the use of a particular splice site is suppressed.

Pre-mRNA splicing is catalyzed by the spliceosome, a dynamic complex composed of small nuclear RNAs (snRNA) and their associated proteins [7]. The assembly of the spliceosome involves sequential binding and release of small nuclear ribonucleo-proteins (snRNPs) and protein factors. It also requires specific formation and disruption of RNA–RNA, protein–RNA and protein–protein interactions. Fundamental to the initiation of a splicing event is the recognition by the spliceosome of sequence-specific splice sites (SS) at the intron–exon borders and the assembly of small nuclear ribonucleic proteins (snRNPs) on the pre-mRNA sequences (Figure 1).

**Figure 2:**
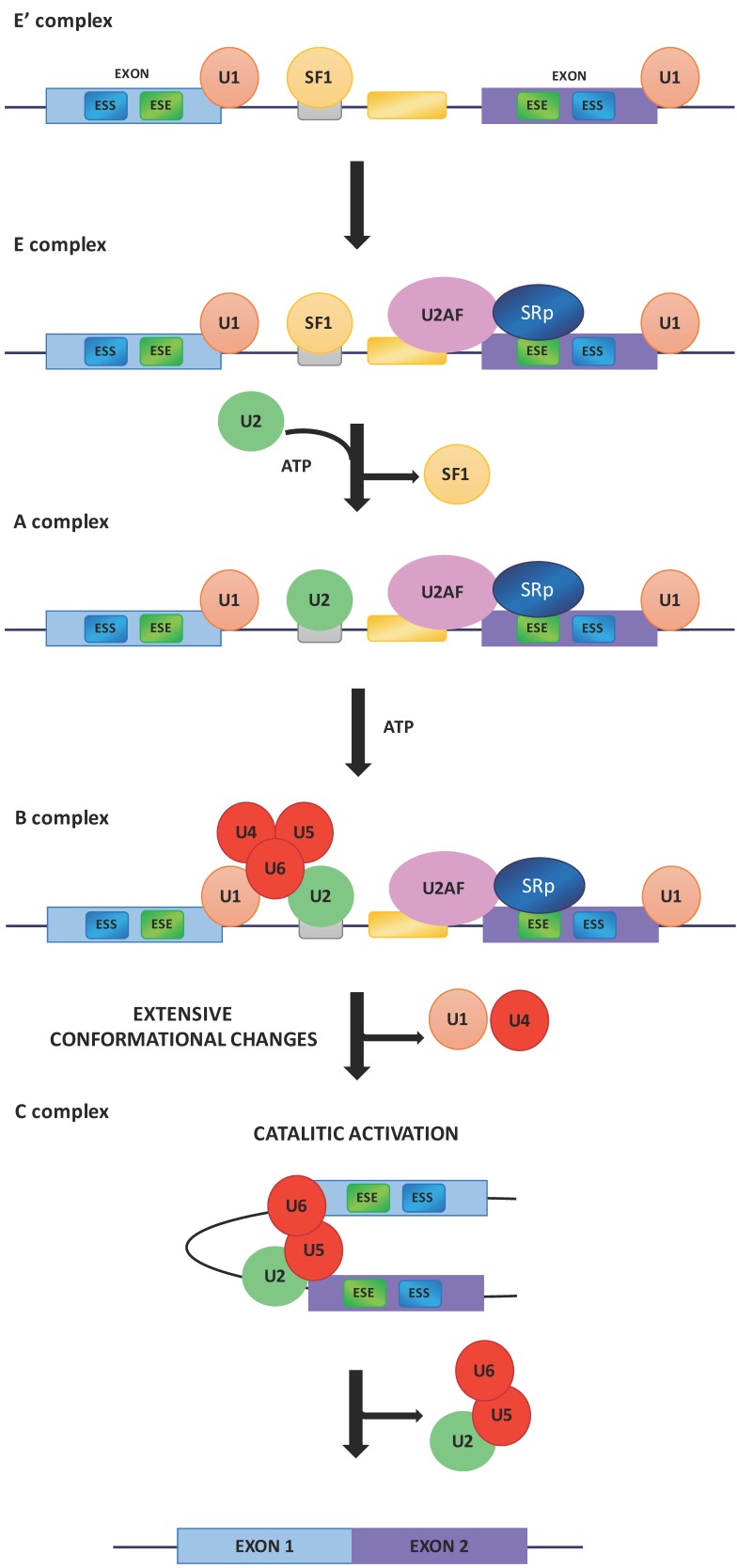
Basic mechanism of spliceosome assembly. ESS, exonic splicing silencer; ESE, exonic splicing enhancer; U1, U1 small nuclear ribonucleoprotein; SF1, splicing factor 1; U2AF, U2 auxiliary factor; SRp, SR (Ser-Arg) proteins; U2, small nuclear ribonucleoprotein; ATP, adenosine triphosphate; U4/U5/U6, U4/U5/U6 small nuclear ribonucleoprotein complex.

There is a great deal of evidence demonstrating that the splicing is generally a co-transcriptional event [8,9,10] and that the two processes are coupled and influence each other [11]. In addition, transcription enhancers [12], transcription factors [13], co-activators [14], chromatin remodelers [15] as well as altering factors for chromatin structure [16,17,18] can also affect the modulation of alternative splicing. The recognition and use of specific splice sites by the splicing machinery is affected by different *cis*-acting elements as intronic or exonic splicing enhancers (ISEs or ESEs) and intronic or exonic splicing silencers (ISSs or ESSs) (Figure 1) [19]. Both ISEs/ESEs and ISSs/ESSs are very conserved structures and distributed throughout the genome [7]. Commonly, ISEs/ESEs and ISSs/ESSs exert their effects throughout the binding of regulatory SR (Ser-Arg) proteins (SRp) and hnRNPs [20,21]. The SR proteins are characterized by the presence of an RS domain, a region rich in arginine–serine dipeptides, and one or two N-terminal RNA recognition motifs (RRMs) that enable its interaction with pre-mRNA [22]. The SR proteins are also involved in the assembly of the spliceosome machinery [7] operating in both alternative and constitutive pre-mRNA splicing [23,24]. The use of different splice sites depends on the link between the SR proteins and the exonic or intronic *cis*-acting factors. For example, the SR protein binding sites of the ESE promote the choice of particular splice sites, whereas intronic SR binding sites suppress the splice site selection [25,26]. The splicing process begins with the base pairing of U1 snRNP to the 5′ splice site of the pre-mRNA and the binding of splicing factor 1 (SF1) to the branch point sequence (BPS) [27,28,29] in an ATP-independent manner to form the E′ complex (Figure 2).

The E′ complex is subsequently converted into the early complex (E complex) by the recruitment of U2 auxiliary factor (U2AF), a dimer of 65 and 35 kDa subunits, to the polypyrimidine tract and 3′ terminal AG [30,31]. SR proteins bind the ESEs and contact U2AF, U1 snRNP and the branch point [32,33,34,35,36,37]. The ATP-independent E complex is then converted into the ATP-dependent pre-spliceosome, known as A complex, by the replacement of SF1 with U2 snRNP at the branch point sequence. The recruitment of the U4/U6–U5 tri-snRNPs leads to the formation of the precursor B complex, which consists of all the necessary spliceosomal subunits that carry out pre-mRNA splicing. After extensive conformational changes and the detachment of U1 and U4 snRNPs, the B complex forms the catalytically active C complex spliceosome (Figure 2) [31].

## 3. Role and Type of Alternative Splicing

Alternative splicing results in the generation of numerous different isoforms from a single gene and can be considered a source for discrepancy between estimated numbers of protein-coding genes in the human genome [38]. Literature has established that nearly 95% percent of human genes undergo alternative splicing events [38,39,40], highlighting the potentially central role in determining phenotypic complexity within and between individuals and among populations. In physiological conditions, alternative splicing allows organisms to properly respond to variations in the environment by addressing the expression of different and space-temporal proper isoforms.

**Figure 3:**
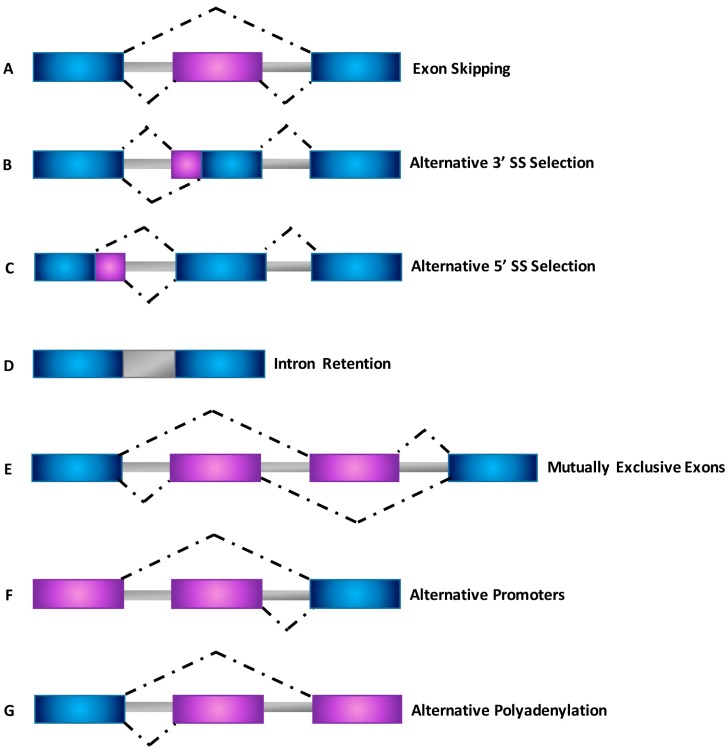
Schematic representation of different types of alternative splicing events. (**A**) Exon skipping; (**B**) Alternative 3’ splice site selection; (**C**) Alternative 5’ splice site selection; (**D**) Intron retention; (**E**) Mutually exclusive exons; (**F**) Alternative promoters; (**G**) Alternative polyadenylation. Dot lines indicate alternative splicing options.

Systematic analyses of expressed sequence tags (ESTs) and cDNAs showed that there are several different types of AS events that are expressed with greater complexity in mammalian species [41] (Figure 3).

The most common type of AS, accounting for nearly 40% of events in higher eukaryotes [42,43], is exon skipping, in which a cassette exon is spliced out of the transcript together with its flanking introns (Figure 3A). Different examples of AS include alternative 5′ splice site (5’ SS) and *3′* splice site (3’ SS) selection within exon sequences in which two or more splice sites are recognized at one end of an exon (Figure 3B,C) [44]. Intron retention (Figure 3D) is the rarest AS event in vertebrates and invertebrates, and occurs when an intron is retained in the mature mRNA transcript [42,43,44,45,46,47]. Finally, mutually exclusive exons (Figure 3E), alternative promoter usage (Figure 3F) and alternative polyadenylation (Figure 3G) are regarded as uncommon events responsible for the generation of alternative spliced transcripts [27,46,48].

## 4. Alternative Splicing (AS) and Diseases

Changes in alternative splicing patterns have an essential role in normal development, cell differentiation and occurs in response to physiological stimuli. AS is one of the main control mechanisms for the development of a cellular phenotype and, in various diseases, it is clearly deregulated. When aberrant splicing events occur, the generation of new splice variants contributes to multiple aspects of different human pathologies such as genetic disorders, neurodegenerative diseases or cancer, but it can also lead to an increased susceptibility to particular pathological conditions during one’s lifetime [49,50].

### 4.1. AS and Genetic Disorders

Many genetic disorders are inherited and at least 10% are caused by mutations affecting the essential splicing *cis*-elements at the 5′ and 3′ SS’s as well as the exonic and intronic splicing *cis*-elements [51,52,53]. Acquired mutations in these regulatory elements have been reported to be involved in the pathogenesis of several diseases. For example, splice site mutations in the *DMD* gene, which encodes for dystrophin protein, results in the loss of dystrophin function that leads to Duchenne muscular dystrophy (DMD, OMIM #310200) [54,55]. Another example involves Myotonic Dystrophy type 1 (DM1; OMIM #160900), an autosomal dominant inherited disease affecting multiple tissues with symptoms that span from myotonia, progressive muscle wasting, cardiac arrhythmias to insulin resistance [56]. DM1 is caused by an expanded CUG repeat (CUG^exp^) sequence at the 3’ UTR of the dystrophia myotonica protein kinase (*DMPK*) gene [57,58]. The pathology of DM1 can be primarily attributed to the dysregulation of two RNA-binding protein families: muscleblind-like (MBNL) and CUGBP and Elav-like family members (CELF). MBNL1 and CELF1 are both essential for a normal splicing regulation of many pre-mRNAs during development [59,60]. In this scenario, the disruption of their functions by CUG^exp^ RNA leads to missplicing of a number of genes such as the muscle-specific chloride channel (*CLCN1*) gene, bridging integrator 1 (*BIN1*) gene, insulin receptor (*IR*) gene, pyruvate kinase M (*PKM*) gene, and cardiac troponin T (*TNNT2*) gene, all of which represent the prominent characteristics of the disease [61]. The alteration of *IR* and *CLCN1* pre-mRNA processing points toward the hypothesis of a spliceopathy, which leads to an inadequate expression of isoforms for a particular tissue or developmental stage [62]. Similarly, a CCTG expansion in intron 1 of the zinc finger protein 9 (*ZNF9*) gene causes Myotonic Dystrophy type 2 (DM2; OMIM #602668) [63]. Although strikingly similar, DM1 and DM2 phenotypes are not identical. The clinical and molecular analogies of DM1 and DM2 demonstrate the multisystem effects of CUG and CCUG expansions, respectively. However, downstream differences in the expression of the CUG-versus CCUG-containing transcripts or epigenetic modification at DM1 and DM2 loci could be responsible, on the other hand, for the clinical distinctions between the two diseases [64].

Alternative splicing is particularly important in the brain as observed by the alterations of factors involved in the regulation of splicing events responsible for a range of neurological disorders spanning from spinal muscular atrophy (SMA; OMIM #253300) to amyotrophic lateral sclerosis (ALS) [65]. SMA is an autosomal recessive neuromuscular disorder caused by the deletion or mutation of Survival Motor Neuron 1 (*SMN1*) gene [66]. In humans, the *SMN* gene that codes for snRNP assembly factor is located on chromosome 5q13 and is identified as *SMN1* (telomeric) and *SMN2* (centromeric) [67]. *SMN2* gene is almost identical to the *SMN1* gene but it is unable to produce sufficient amounts of full-length transcripts because of a C to T transition in the coding exon 7. Consequentially, alternative splicing then creates a truncated protein (SMNΔExon7) lacking exon 7 and resulting in a product that is unstable, is non-functional and degrades rapidly [67,68,69]. However, *SMN2* produces low levels (5%–10%) of the full-length SMN protein that are sufficient for survival but still result in SMA syndrome [70]. The functional analysis of SMN revealed a link to the pre-mRNA processing spliceosome. Literature implicates SMN in the assembly of the Sm-class of U snRNPs and therefore also plays a role in the formation of the functional spliceosome [71,72,73,74,75]. It is clear that even slight changes in the spliceosomal machinery formation and regulation represent a critical challenge point for the development of many genetic disorders.

### 4.2. AS and Cancer

Aberrant splicing variants are known to be highly expressed in cancer, thereby contributing to multiple aspects of the pathology throughout tumorigenesis and development of resistance to therapeutic treatment [76,77,78]. Many cancer-associated splicing isoforms are expressed during embryonic development but not in normal adult tissues, whereas other cancer-associated splicing isoforms are entirely new transcripts [79]. An illustrative example of isoform shift in different tumor stages involves the *CPT1* (Carnitine Palmitoyltransferase 1) gene that physiologically resides at the outer mitochondrial membrane of normal cells and represents a site for intracellular regulation of lipid metabolism by transporting long-chain fatty acids into mitochondria for β-oxidation (together with CPT2 and Carnitine/Acyl-Carnitine Translocase) [80,81]. In cancer tissues, the splice isoform CPT1A variant 2 (CPT1Av2) is strongly expressed only in the nucleus of tumoral cells and identified as a new partner of histone deacetylase (HDAC), involved in epigenetic transcriptional regulation. The knockdown of *CPTIAv2* modulates histone acetylation and induces apoptosis, strongly affecting the expression of cancer-relevant genes and demonstrating an alternative function of this metabolic variant in tumor insurgence and progression [82]. During neoplastic transformation, cells acquire characteristic features such as sustenance of proliferative signaling, resistance to cell death, the ability to invade normal tissues, replicative immortality, induction of angiogenesis and the ability to avoid the immune system [83]. Many of these traits can be directly linked to aberrant gene expression regulation resulting from dysregulation of alternative splicing patterns during cancer progression. Notable genes that undergo alternative splicing and are known to be altered during cancer development include tumor suppressor genes, such as *p53* and *BRCA1*, and oncogenes, such as *Ras* and *EGFR* [84,85,86,87,88]. A critical aspect of tumorigenesis is uncontrolled cell proliferation and the ability to grow in absence of external growth stimuli [89,90,91]. Recently, genes involved in lipid metabolism have been found highly enriched in cancer tissues. In this scenario, LDLs are a common hub between cancer and metabolic gene networks suggesting the importance of lipid metabolism during cellular transformation. Indeed, oxidized LDLs can cause transformation of MCF-10A cells (human epithelial mammary gland cell line) lacking ER-Src in a manner that depends on NF-κB.

### 4.3. AS in the Diseased Heart

Alternative splicing plays a crucial role in development, homeostasis and disease of the heart as shown by a large-scale splicing microarray approach that discovered 63 alternative splicing events in the development of mouse heart [60]. In humans, postnatal heart development is accompanied by major alternative splicing changes as well [92]. One of the first documented examples involves the alternative splicing factor ASF/SF2 (or SRSF1) [93], which is a ubiquitously expressed SR protein that acts as a constitutive and alternative splicing regulator. *ASF/SF2* knockout mice are embryonically lethal, and conditional heart-specific ablation causes a hypercontractile cardiac phenotype caused by a defect in Ca^2+^ handling. Deletion of *ASF/SF2* leads to missplicing of several gene products, including Ca^2+^/calmodulin-dependent protein kinase (CamkIIδ), cardiac troponin T (cTnT), and LIM-domain binding 3 (LDB3). Missplicing of CamkIIδ in *ASF/SF2* knockout hearts results in disturbed Ca^2+^ handling and severe excitation–contraction coupling defects, leading to dilated cardiomyopathy (DCM) [94,95]. SR-rich splicing factor 2 (SRSF2 or SC35) is another ubiquitously expressed SR protein whose systemic deletion in mice results in embryonic lethality before the onset of cardiogenesis. The generation of heart-specific knockout of *SC35* uncovered the role of SC35 in the heart, as these mice developed cardiac hypertrophy and DCM at five to six weeks of age. The disease phenotype is associated with a downregulation of ryanodin receptor 2 (Ryr2) in the *SC35* knockout hearts [96]. Ryanodine receptor 2 is responsible for the release of Ca^2+^ from intracellular stores during excitation−contraction coupling in both cardiac and skeletal muscle. Mutations in *RyR2* are associated with human disorders such as catecholaminergic polymorphic ventricular tachycardia [97]. Studies on alternative splicing changes in the heart have revealed large differences during development specific to disease states. Rather than reactivation of selected fetal genes, several fetal splice isoforms are re-expressed in the stressed or diseased heart such as the fetal isoforms of the sarcomeric proteins Titin and Myomesin [98,99,100,101]. The splicing of four key sarcomeric genes, troponin T2 (*TNNT2*), *TNNI3*, *MYH7*, and *FLNC* were significantly altered in human ischemic cardiomyopathy, DCM, and aortic stenosis [101]. Until now, there have only been a few examples of mutations in the splice sites themselves that are shown to directly cause human heart diseases [95]. One of the first splice site mutations that has been reported to result in heart disease is a G>A point mutation that disrupts the 5′ splice site of exon 15 of cardiac *TNNT2* gene and leads to truncated mRNA variants. In addition, this mutation causes not only the exon 15 skipping but also to the activation of a cryptic splice site in exon 15, resulting in the generation of a second aberrant splicing product of TNNT2. Consequently, sarcomeric contractions are impaired and hypertrophic cardiomyopathy arises [102]. A different mutation, which also involves creating a new splice site, involves *SCN5A* gene that encodes the α subunit of the cardiac sodium channel. In this example, a 4 bp insertion in exon 27 creates a cryptic splice site that causes a deletion of 96 bp in the transcript resulting in the loss of key domains of SCN5A protein. The mutant channel fails to express any sodium current and leads to Brugada Syndrome (OMIM #601144), a heart disease that is characterized by an abnormal electrocardiogram (ECG) and increased risk of sudden death [103].

## 5. Is Alternative Splicing a Potential Therapeutic Target?

To the best of our knowledge, over 2000 splicing mutations are known, involving 303 genes implicated in 370 diseases [104]. As such, the field of research concerning AS modulators can develop novel and promising therapeutic strategies that focus on switching the splicing of a “risk” isoform, identified as harmful and promoting the pathological process, towards a “protective” isoform [105]. Different approaches have been explored, including antisense oligonucleotides (ASOs), splicing-switching oligonucleotides (SSOs), antisense snRNAs, RNA interference (RNAi) and small molecules [106,107] to modify the splicing pattern of a mutant pre-mRNA or eliminate an mRNA that bears a disease-causing mutation. So far, the most utilized strategy involves ASOs or SSOs. Generally, the strategy behind ASOs is to hybridize and block one or more sequences in the pre-mRNA target that are critical for a particular splicing event, and cause the splicing machinery to select an alternative pattern whose outcome is more favorable. To be effective, the ASO’s target to its mRNA needs to be accessible in the native RNP and it must bind in a specific and selective manner [108]. ASOs in conjunction with various backbone chemistries have been developed in order to improve affinity, boost stability in the circulation and in target cells, and enhance cell penetration and nuclear accumulation [109]. ASOs and SSOs have been proven to be very promising [53], for example in spinal muscular atrophy (SMA) and myotonic dystrophy type 1 (DM1). A large-scale analysis of ASOs targeting the intron adjacent to the exon 7 of *SMN2* gene highlighted specific ASOs that drastically promoted exon 7 inclusion thus leading to the production of a full-length functional SMN protein that is lacking in SMA. Using a mouse model, a single intracerebroventricular (ICV) injection of ASO-10-27 showed a dose-dependent increase of exon 7 inclusion, SMN protein synthesis as well as an improved muscle physiology and motor function [108]. Importantly, systemic delivery of ASO-10-27 in neonatal SMA mice enhanced SMN levels in peripheral tissues as well as in the central nervous system determining an increased skeletal muscle function and a significant rise in survival rate [110,111]. 

MBNL1 loss of function is responsible for more than 80% of splicing pathology in DM1 mice models [112,113]. DM1 results from the buildup of toxic gain-of-function RNA, which would be the main target for therapeutic strategies that involve targeting it to cause its degradation or covering the RNA in order to render its binding sites unavailable for interaction with RNA-binding proteins. In this scenario, one of the first applications of ASOs was the use of a retrovirus that expressed an ASO complementary to (CUG)_13_ repetition of *DMPK* mRNA [114]. As consequence, DM1 myoblasts expressing the ASO showed significant improvement in several characteristic markers of the disease such as myotube formation, fusion index, and glucose uptake. However, the use of this ASO resulted in the reduction of both repeat containing and normal *DMPK* transcripts. The development of CAG7, a modified ASO complementary to CUG repeats [115] and then the development of CAG25 [116], demonstrated the efficacy of ASO-mediated silencing for DM1 treatment. Indeed, treatment of HSA^LR^ mice (mouse model for DM1) with CAG25 resulted in a significant reduction of RNA foci, reduction of CUG^exp^ RNA, redistribution of MBNL1, rescue of missplicing and reduction of myotonia levels.

Despite these promising results involving SSO and ASO, the use of small molecules splicing modulators (smSMs) continue to be the an attractive therapeutic approach. Small molecule-based strategies involve the sterical inihibition of the interaction between the CUG^exp^ RNA and the RNA-binding proteins. The first small molecule with a demonstrated efficacy in DM1 cell culture models was pentamidine, which was discovered in a large screen for the identification of nucleic acid-binding compounds capable of disrupting the binding of MBNL1 to CUG^exp^ RNA [117]. Pentamidine treatment fully rescued splicing defects in a DM1 cell culture model.

The number of smSMs that have been shown to affect splicing is growing. An interesting example is represented by amiloride, a long-used diuretic [105] that has been found to potently affect splicing of several genes involved in apoptosis and subsequently able to decrease tumor growth in animal models [118]. Recently, a small molecule compound, SRPIN340, that inhibits major regulators of AS through SR-protein phosphorylation, SRPK1 and SRPK2 [119] has been shown to inhibit VEGF splicing and angiogenesis in a model of ocular neovascularization [120] as well as melanoma xenograft growth [121] and orthotopic prostate [122] cancer mouse models. However, specificity remains an important obstacle for treatment involving ASOs and smSMs. A recent paper reported the development of smSMs affecting the SMN splicing and causing the attenuation of spinal muscular atrophy. An RNA-seq analysis confirmed the specificity of the treatment and it was observed that just few splice junctions were affected [123], showing that specificity in splicing therapeutics using small molecules is a challenge, but it can be accomplished.

## 6. LOX-1 Receptor

The lectin-like oxidized low-density lipoprotein receptor 1 (LOX-1) is a wide-ranging type II transmembrane protein (52 kDa) that belongs to the C-type lectin family. It is found mainly on the surface of endothelial cells [124] but it is also expressed by macrophages, smooth muscle cells, platelets and dendritic cells [125,126,127,128]. LOX-1 is the main endothelial receptor for oxidized LDLs (ox-LDLs) [124], but it also binds multiple ligands, including acetylated LDLs, polyanionic chemicals, anionic phospholipids, cellular ligands (CRPs) [129,130] and Gram-positive and Gram-negative bacteria including *Staphylococcus aureus* and *Escherichia coli*, in a mechanism similar to that of class A scavenger receptors [131]. The structure of LOX-1 receptor consists of four domains: the N-terminal cytoplasmic domain, the transmembrane domain, the NECK domain and the extracellular C-terminal domain called C-type lectin-like domain (CTLD). CTLD is a ligand-binding domain while the NECK domain is important to maintaining the dimer structure [132].

On the cell surface, LOX-1 appears as a heart-shaped receptor constituted by three homodimers, which surround a central hydrophobic tunnel that extends through the entire molecule. Each LOX-1 homodimer acts as a structural unit to form noncovalent bound tetramers and hexamers [133]. Studies of site-directed mutagenesis indicated the key role of Cys140 in the formation of disulfide-linked LOX-1 dimers [133]. Additional chacterization revealed a drastic reduction in receptor-binding activity by introducing single amino acid substitutions at the dimerization interface suggesting a specific conformational arrangement is crucial for ligand binding [132]. The hydrophobic tunnel was functionally studied by site-directed mutagenesis and X-ray crystallography [134]. These studies showed that the replacement of Ile149, at the gate of the channel resulted in an occlusion restricting the docking of ligands thus implicating the central hydrophobic tunnel as a key functional domain that is critical for the recognition of modified LDLs [134]. The specific mechanism of ox-LDL internalization by LOX-1 was described by several studies. A site-directed mutagenesis study in HeLa cells transfected with LOX-1 cDNA containing an engineered FLAG tag identified a tripeptide conserved motif (DDL) in a position proximal to the N-terminal, as well as the cytoplasmic domain of LOX-1 that is responsible for aiding ox-LDL internalization; this mechanism was also modulated by dynamin-2 [135,136]. A structural modeling of the LOX-1 cytoplasmic domain revealed that the DDL tripeptide motif, implicated in ox-LDL endocytosis, is part of a curved β-pleated sheet structure. A triple alanine substitution of the DDL motif substantially reduced endocytosis of ox-LDLs. Moreover, transplantation of the LOX-1 cytoplasmic domain into a transferrin receptor reporter conferred efficient endocytosis confirming that the endocytic motif DDL within the LOX-1 cytoplasmic domain is needed to ensure efficient internalization of pro-atherogenic ox-LDL particles [136]. In human coronary artery endothelial cells, LOX-1 recognizes and internalizes its ligands through the caveolae/raft-dependent endocytosis pathway. Furthermore, LOX-1 was palmitoylated and both the amino acide residues Cys36 and Cys46 were essential for the recruitment of LOX-1 into raft micro-domains and for its function [137].

## 7. OLR1 Alternative Splicing: One Gene—Three Proteins

**Figure 4:**
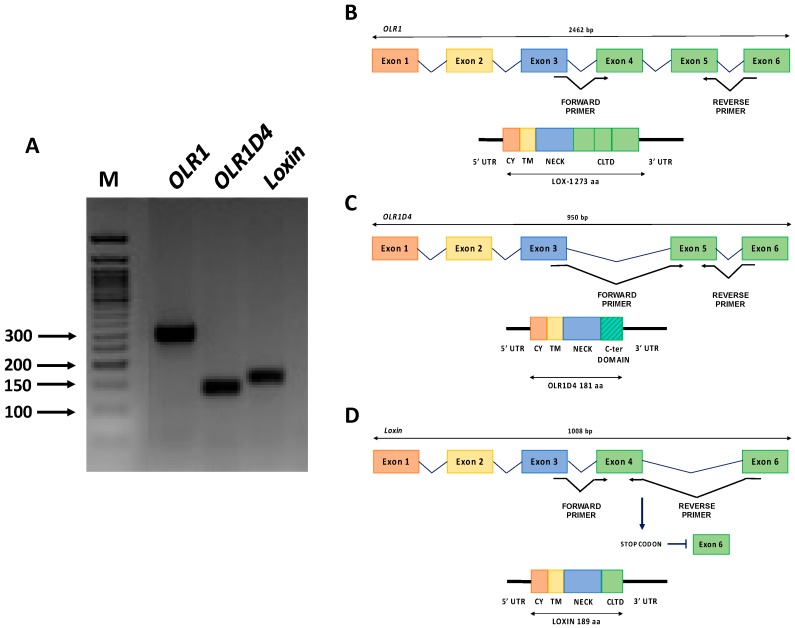
*OLR1* gene isoforms. (**A**) *OLR1* (290 bp), *OLR1D4* (140 bp) and *Loxin* (160 bp) isoforms pattern on agarose gel. M (Marker): 50 bp DNA ladder (BioLabs); (**B**) Top: intron-exon sequence of *OLR1* full-length isoform. Bottom: schematic representation of the protein product; (**C**) Top: intron-exon sequence of *OLR1D4* isoform. Bottom: schematic representation of the predicted protein OLR1D4; and (**D**) Top: intron-exon sequence of *Loxin* isoform. Bottom: schematic representation of the predicted protein LOXIN. The position of the primers pairs specific for each isoform is also shown in the figure. 5’ UTR, 5’ untranslated region; CY, cytoplasmic domain; TM, transmembrane domain; NECK, neck domain; CLTD, C-terminal lectin type domain; 3’ UTR, 3’ untranslated region.

LOX-1 is encoded by the single-copy *OLR1* gene (OMIM #602601) mapped on the p12.3–p13.2 region of human chromosome 12 [138]. *OLR1* maps within the Natural Killer (NK) gene complex and LOX-1 receptor is formed by four protein domains as well as the NK receptors (Figure 4). The gene structure and promoter region of *OLR1* were deeply investigated [138] and a wide variety of stimuli were found to upregulate *OLR1* at the transcriptional level, including Angiotensin II (Ang II), an integral regulator of the renin-angiotensin system, that upregulates *OLR1* transcription through an NF-κB-binding motif located at −2158 nt. Ox-LDLs also upregulate *OLR1* via an Oct-1 motif located at −1556 nt [139,140]. The ox-LDL/LOX-1 binding activates a positive feedback loop that further increases LOX-1 expression via an unknown regulatory governor. *OLR1* gene spans more than 7000 bp and consists of six exons and five introns. Exons 1–5 ranges from 102 to 246 bp, whereas exon 6 is considerably longer spanning 1721 bp. Exon 1 encodes the 5′-untranslated region (5’ UTR) and the cytoplasmic domain (CY), exon 2 encodes the remainder of the cytoplasmic domain and the transmembrane domain (TM), exon 3 encodes the neck domain (NECK) and exons 4–6 encode the C-terminal lectin type domain (CLTD) and the 3′-untraslated region (3’ UTR) (Figure 4) [124,138].

With regards to alternative splicing, *OLR1* generates three splice variants (Figure 4A).

**Figure 5:**
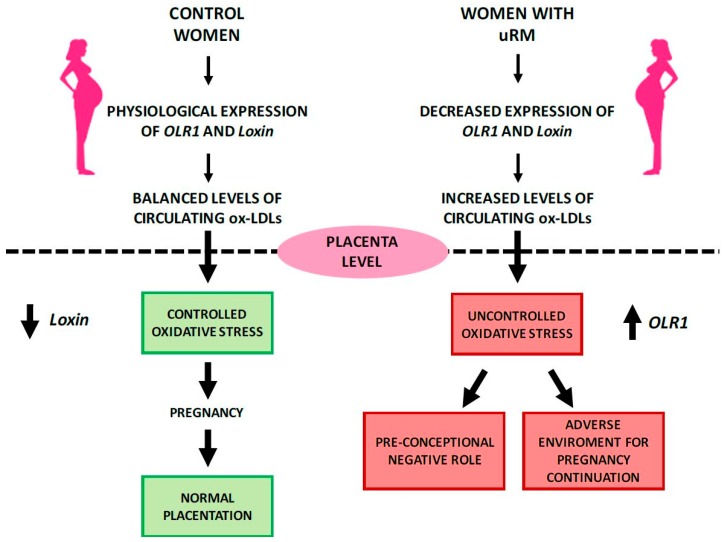
Potential pathophysiological action of *OLR1* gene in physiological and unexplained recurrent miscarriages (uRM) pregnancies (modified from Bruno et al., in press, [147]).

Transcript variant 1 (NM_002543) corresponds to the full-length *OLR1* and is the longest splice isoform spanning 2462 bp and all six exons (Figure 4B). In contrast, transcript variant 2 (NM_001172632), subsequently referred to as *OLR1D4*, is the shortest splice isoform spanning 950 bp and lacking exon 4. It results in a putative LOX-1D4 protein which is shorter than LOX-1 and has a distinct C-terminus that lacks the ligand binding and recognition domains (Figure 4C). Transcript variant 3 (NM_001172633), referred to as *Loxin*, is 1008 bp long without exon 5; it presents a premature stop codon and consequent premature termination generating a predicted protein LOXIN that is missing two-thirds of the lectin-like domain (CTLD) (aa188-aa273) (Figure 4D). It has been well established that truncation of the CTLD of LOX-1 abrogated ox-LDL-binding activity. Specifically, the deletion of the last ten amino acid residues at the C-terminus (261–270) is enough to disrupt the ox-LDL-binding activity. In addition, the substitution of Lys262 and/or Lys263 with alanine residues reduces this activity. Studies of serial-deletion showed that residues up to 265 are necessary to guarantee a minimum binding activity, even though the deletion of the last three C-terminal residues (268–270) seems not to alter full binding activity. In conclusion, a conserved C-terminal lectin-like domain, particularly the basic amino acid pair, is essential for ox-LDL binding [141]. LOXIN dimerizes and sequesters full-length LOX-1 in the plasma membrane preventing the cell from binding and being damaged by ox-LDLs. The LOXIN/LOX-1 heterodimers result in the disruption of the functional properties of LOX-1 and increase the resistance of cells to ox-LDL-induced apoptosis [142]. A recent study performed in human endothelial progenitor cells (hEPC), demonstrated that overexpression of LOXIN protected hEPC from ox-LDL-induced apoptosis and that transduced LOXIN localized to the plasma membrane and blocked ox-LDL uptake mediated by LOX-1 [143]. Furthermore, LOX-1 is highly expressed in vascularized tissues such as placenta [128], which prompted its potentially crucial role in placental function during the earliest stages of pregnancy [144]. Literature data indicate that systemic and placental oxidative stress may have a potential key role in the etiology of recurrent miscarriage (RM) [145,146,147]. Therefore, we conducted a pilot study aimed to characterize *OLR1* and *Loxin* peripheral expression pattern in unexplained recurrent miscarriage (uRM) [147]. Our results show that during the pre-conceptional period, uRM women express significantly lower peripheral levels of both *OLR1* and *Loxin* isoforms compared to control women. In the first trimester of uRM pregnancies, however, the peripheral *OLR1* expression increases significantly. This study is the first to suggest that a different regulation of mRNA isoform expression, probably driven by an enhanced oxidative stress, is playing out at the beginning of physiological and RM pregnancies (Figure 5).

**Figure 6:**
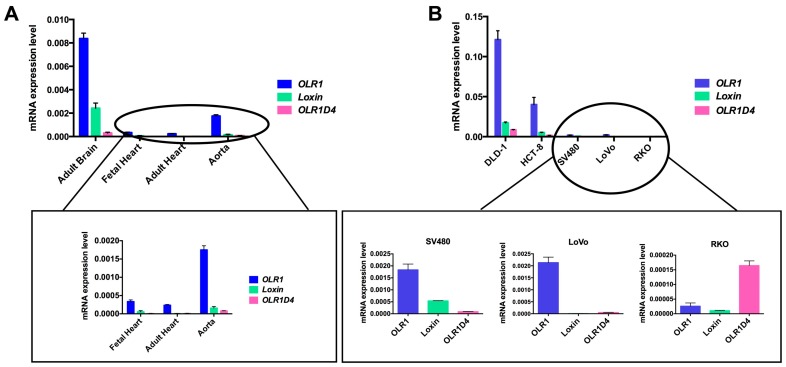
*OLR1* splice variants expression in human tissues (**A**) and colorectal cancer cell lines (**B**). On the *Y*-axis are reported the values of 2^−∆*C*t^ that represent the expression level of each isoform normalized to β-actin in the different tissue and cells analyzed.

To the best of our knowledge, no data on *OLR1D4* isoform and its putative encoded protein LOX-1D4 is available. To elucidate the expression pattern of this less-known splice isoform, we performed an isoform specific quantitative real time-PCR (qRT-PCR) on different human tissues (adult brain, adult and fetal heart, aorta) and on colon cancer cell lines (DLD1, HCT-8, LoVo, SV480, and RKO). Specifically, we obtained total RNA from tissues and cell lines using 1 mL of Trizol Reagent (Ambion, Foster City, CA, USA). One 1 µg of total RNA was reverse transcribed into cDNA using the High-Capacity cDNA Reverse Transcription Kit (Thermo Fisher Scientific, Boston, MA, USA). TaqMan Gene Expression Assay with primers and probes specific for each isoform were used to assess *OLR1* isoform expression (*OLR1* Hs01562594_g1, *Loxin* Hs01552596_m1, *OLR1D4* Hs01598314_m1, Thermo Fisher Scientific, Boston, MA, USA). For each sample, we obtained an amplification plot in which the *C*t (Threshold Cycle) is proportionally inverse to the expression. The expression of each target was calculated through normalization with human β-actin gene as endogenous control (*C*_tGene target_ − *C*_tHK_ = Δ*C*_t_) and then using the 2^−^^∆*C*t^ formula. Expression analysis was performed in triplicate for each sample. The results obtained show that *OLR1* splice variant is the main expressed isoform (Figure 6).

Among human tissues, the adult brain displayed the highest *OLR1* expression level in addition to relatively high expression of *Loxin* and *OLR1D4* (Figure 6A). In the human aorta, *OLR1* expression is four-fold lower than in brain. In addition, both adult and fetal heart tissues exhibit a very low *OLR1* splice variant expression.

Among colon cancer cell lines, DLD-1 exhibits the highest *OLR1* expression and also a significant expression of *Loxin* and *OLR1D4* (Figure 6B). However, *OLR1D4* isoform is the least represented in all the types of tissue studied except for RKO cell lines (Figure 6B).

We believe this peculiar profile of *OLR1D4* in RKO cells is worthy of a deeper investigation.

Murine *Olr1* gene is also subjected to AS that generates two splicing isoforms: *D3D5Olr1* (GenBank ID HQ603086), with a length of 570 bp, lacking exons 3, 4 and 5, and *D2D5Olr1* (GenBank ID HQ603085), 468 bp long missing exons 2, 3, 4 and 5. The putative protein product of the isoform *D3D5Olr1* (D3D5Lox-1) lacks othe “neck” domain, while the D2D5Lox-1 is devoid of eight amino acids of the cytoplasmic domain and of the entire transmembrane domain and “neck” domain, this results in an absence of D2D5Lox-1 localization on the plasma membrane. Notably, both isoforms are mainly expressed in the embryonic stage at 7.5, 8.5 and 9.5 dpc and not in adults [148]. The aforementioned data that shows the overlapping expression pattern of *OLR1* and its splice forms both in humans and in mice is intriguing as it suggests that, if co-expressed, alternatively spliced isoforms may act as negative regulators [149].

## 8. *OLR1* Splicing Regulation

There is a growing consensus that splicing efficiency may be a significant contributor to phenotypic variability [150]. In addition, splicing and alternative splicing are shown to significantly impact phenotypes and they contribute to severity and susceptibility to human diseases [3,151].

Given the density of the splicing code, consisting of consensus splice site sequences positioned at exon–intron boundaries and intronic and exonic splicing enhancer and silencer elements along the genome, it is not surprising that normal genetic variations such as single nucleotide polymorphisms (SNPs) can be attributed to splicing efficiency and alter either the total output of a gene or the ratio of alternatively spliced variants [3]. A large-scale study of human genetic variations within and around the exons of more than 300 genes highlights that 1.3% of the SNPs identified were located in the consensus splice sites [152].

To this respect, seven different single nucleotide polymorphisms (SNPs), situated within intron 4 (rs3736232 G>C; rs3736234 C>T; rs3736235 A>G), intron 5 (rs17174597 A>G; rs13306593 G>T) and the 3′ UTR (rs1050283 C>T) of *OLR1* gene (Table 1) are located in a linkage disequilibrium (LD) block strongly associated with an elevated risk to develop coronary arteries disease (CAD) and myocardial infarction (MI) [153,154,155].

**Figure 7:**
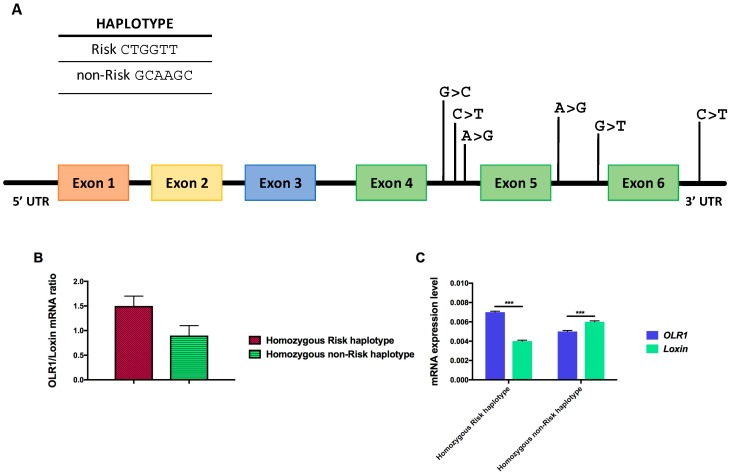
The seven SNPs in the LD block of *OLR1* gene modulate *OLR1* and *Loxin* expression. (**A**) The exon-intron structure of *OLR1* gene is shown along with the position of the SNPs comprised in the LD block. Top: genomic configuration of “Risk” (CTGGTT) and “non-Risk” (GCAAGC) haplotype. (**B**) Bar graphs show the relative amount of the two isoforms (both normalized to β-actin) expressed as a ratio of *OLR1* to *Loxin* isoform. (**C**) Bar graphs show the relative amount of *OLR1* and *Loxin* isoforms depending on the specific haplotype. On the *Y*-axis are reported 2^−^^∆*C*t^ values that represent the expression level of each isoform in the analyzed Periferal Blood Mononuclear Cells PBMCs. Data are expressed as mean ± standard deviation. Student *t* Test; *** *p* ≤ 0.0005.

The genotypic configuration of these SNPs can generate two different haplotypes; one is called “Risk” (CTGGTT) and related to an increased chance of developing myocardial infarction compared to the other one called “non-Risk” (GCAAGC) haplotype (Figure 7A).

An in vitro minigene assay showed that the SNPs located in the LD block regulate the expression level of *Loxin* transcript by modulating the inclusion and skipping of exon 5 from the *OLR1* gene transcript [156]. Isoform-specific quantitative real-time PCR (qRT-PCR) assays performed on total RNA of human monocyte-derived macrophages of CAD patients who are carriers of the “Risk” and “non-Risk” haplotype, demonstrated a significant difference in the *OLR1*/*Loxin* mRNA ratio correlating with their haplotype. For instance, homozygous “Risk” haplotype subjects show a higher *OLR1*/*Loxin* ratio compared with those homozygous for the “non-Risk” haplotype (Figure 7B) [156]. The greater expression of *Loxin* transcript in subjects carrying the “non-Risk” haplotype (Figure 7C) suggests a negative link between levels of *Loxin* and the incidence of MI in humans, portraying *Loxin* as a “protective” splice isoform in cardiovascular pathologies [142,143,156].

In a recent work, Tejedor et al. characterized the contributions of each SNP sequence variation to *OLR1* alternative splicing and identified relevant regulatory sequence motifs and factors using computational, biochemical, mutational and high-throughput screening technologies [157].

In order to assess the contribution of each intronic SNP in the regulation of *OLR1* alternative splicing, Tejedor generated two series of mutant minigene constructs containing the genomic sequences defined “Risk” (CTGGTT) and “non-Risk” (GCAAGC). Sequential site-mutagenesis of each SNP in the risk or non-risk minigenes revealed that mutation of SNPs rs3736234 (intron 4) and rs13306593 (intron 5) displayed the most significant effects on *OLR1* AS regulation. In fact, transition of C>T of rs3736234 in the “non-Risk” minigene resulted in a very significant increase of exon 5 inclusion increasing *OLR1* full-length expression), while the reciprocal change in the “Risk” minigene caused the strongest decrease in exon 5 inclusion corresponding to a higher *Loxin* expression). The prominent effects on *OLR1* splicing regulation from the substitutions of C>T in SNP rs3736234 is not observed with different nucleotide substitutions such as C>G.

This elegant approach clearly demonstrates the involvement of specific SNPs (particularly rs3736234) on *OLR1* alternative splicing regulation.

Moreover, progressively introducing nucleotide changes in the “non-Risk” minigene up to the “Risk” allelic series shows that the strong increase in exon 5 inclusion is caused by the natural variant of SNP rs3736234 and that it can be progressively attenuated by the presence of additional nucleotide variants present in the linkage disequilibrium block until the ratio of inclusion/skipping of exon 5 resembled that of the “Risk” minigene [157].

These results suggest that the difference in exon 5 inclusion/skipping ratio observed in individuals carrying the “Risk” or “non-Risk” haplotypes [156] is the result of the balance between the strong effect of SNP rs3736234 and the compensatory effect of the other SNPs’ mapping in the LD block [157].

In silico analysis identified a variety of potential AS regulatory elements in the region of intron 4 containing the key-regulator SNP rs3736234. In particular, a potential binding site for the SR protein SRSF2 (SC35), partially overlapping with an SRSF1-binding site, was predicted in the “non-Risk” haplotype. When C-rs3736234 nucleotide was replaced by T (present in the “Risk” haplotype), the AS regulator SC35 did not bind. Therefore, a possible scenario emerging from these in silico analyses is that an intronic enhancer element (such as a SRSF1-binding site) promotes exon 5 inclusion in the “Risk” haplotype, while regulatory motifs in adjacent sequences, such as the non-risk C-rs3736234 allele, act as dominant silencer motifs.

A comparative analysis of the genomic sequences around SNP rs3736234 across various vertebrate species revealed conservation of the overlapping human SRSF1 and SRSF2 regulatory elements in different primate species, but not in other vertebrates including mouse or rat [157].

RNA pull-down experiments revealed that SRSF1 actually binds to RNA in the presence of T-rs3736234 (i.e., “Risk”) while the protein does not associate with the same RNA containing C-rs3736234 (i.e., “non-Risk”). This indicates that in individuals bearing the “Risk” allele the levels of SRSF1 could represent an additional risk factor for cardiovascular diseases. SRSF1 plays a key role in the regulation of numerous AS events by frequently working as a component of exonic splicing enhancers and it can be involved in neoplastic transformation and cancer progression [158,159].

Furthermore, the influence of the inflammatory microenvironment and intracellular milieu on the ratio of the different isoforms produced remains to be fully elucidated. It seems that a particular metabolic state in some pathologic conditions, such as tumorigenesis and atherosclerosis, could orchestrate the shift in splice variant and the canonical function of the proteins involved.

The observed involvement of *OLR1* in cancer establishment and progression and the active role of alternative splicing in the generation of splice variants with different effects on disease, implicates a critical role for *OLR1* splice variants in cancer that is yet to be fully understood.

However, it is clear that the modulation of endogenous *OLR1* alternative splicing can represent a potential and promising therapeutic strategy in order to reduce ox-LDL levels and atheroma plaque formation in patients with high susceptibility to cardiovascular disease.

## 9. LOX-1 Signaling and Role in Atherogenesis and Tumorigenesis

### 9.1. Atherogenesis

Atherosclerosis is a slowly developing, age-linked disease of the large- and medium-sized arteries. The primary sites of atherosclerotic plaque formation are the deep intimal layers of large arteries such as the common carotid artery (at the bifurcation), the aorta (at the start of its branches), and the subclavian artery [160,161].

Atherogenesis consists of a sequence of cellular and molecular events that starts with the entrance of lowdensity lipoproteins, which are rich in cholesterol, into the intima of atheroprone sites of arteries and continues with the expression of adhesion molecules [162,163], recruitment of mononuclear cells to the endothelium [164], local activation of leukocytes followed by inflammation [165], lipid accumulation and finally foam cell formation [166]. Accordingly, the major culprit in the generation of atherosclerotic plaque is considered the accumulation of lipids in blood vessels, characteristically low-density lipoproteins. Resistance to lipid clearance and its longer persistence in blood vessels promote chemical oxidation of the existing LDL particles into oxidized LDLs that generate a pro-inflammatory feedback facilitating the formation of additional ox-LDLs and other pro-inflammatory cytokines through the inflammatory pathway [167]. Expression of LOX-1 is especially elevated in human atherosclerotic plaques, particularly at the intimal cellular level as illustrated by LOX-1 upregulation in intimal vascular smooth muscle cell (VSMCs) and in human carotid-plaque macrophages [168]. 

Various studies have shown that binding of ox-LDLs to LOX-1 increases the intracellular level of reactive oxygen species (ROS) such as superoxide anion (O_2_^−^) and hydrogen peroxide (H_2_O_2_). Intracellular nitric oxide (NO), which protects against vascular injury, inflammation and thrombosis, reacts with superoxide anion decreasing cellular NO level. This condition leads to endothelial dysfunction [169].

Numerous interplays exist between inflammation and oxidative stress in the development of endothelial dysfunction. One of them involves NF-κB, an oncogenic protein that regulates the transcription of several genes involved in immune and inflammatory response [170]. NF-κB, which is present in the cytoplasm as a heterodimer sequestered by IκB, can be released when activated and migrate from the cytosol to the nucleus where it binds specific DNA sequences and increases transcription of pro-inflammatory and adhesion molecules, such as TNF-α, ICAM-1, and VCAM-1 [171].

**Figure 8:**
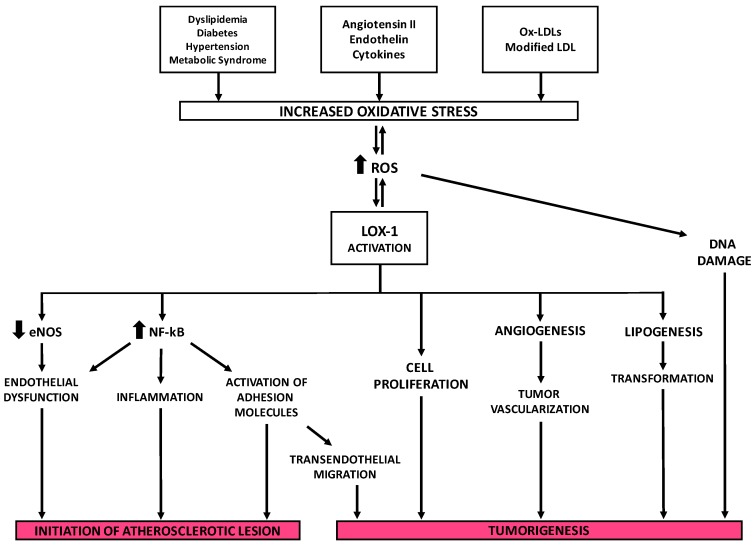
The key role of LOX-1 receptor in the development of atherosclerosis and tumorigenesis. **Thick arrows** indicate up-regulation (**up arrows**) or down-regulation (**down arrows**); **thin arrows** indicate the pathways involved in LOX-1 activation.

NF-κB also plays a role in atherosclerosis. In endothelial cells, smooth muscle cells and fibroblasts, NF-κB is activated by various inflammatory stimuli including ox-LDLs which lead to cell injury [172]. Subsequently, an NF-κB-binding motif in *OLR1* promoter was identified between nt −2247 and −2131 [139]. It has been reported that incubation of an anti-LOX-1 monoclonal antibody inhibited NF-κB activation induced by ox-LDLs suggesting that the binding of ox-LDL to LOX-1 and the consequential formation of ROS are the events that lead to NF-κB activation. Furthermore, LOX-1 expression is found to depend on activation of NF-κB induced by ox-LDLs following the generation of ROS. This produces a feedback circuit in which ox-LDLs induce LOX-1 signaling for the promotion of their pro-inflammatory activity via NF-κB. Demonstratively in endothelial cells, oxidative activation of NF-κB causes changes in cell phenotype and contributes to the initiation of atherosclerotic lesion formation [172] (Figure 8).

### 9.2. Tumorigenesis

Activation of lipid metabolism is a primary event in tumorigenesis and one of the main characteristics of numerous tumors. Epidemiological studies suggest an association between dysregulated metabolism and carcinogenesis. There is a large body of evidence supporting a link among obesity, metabolic syndrome, insulin resistance and inflammation and the increased risk of cancer [170,173,174]. Metabolic syndrome is characterized by elevated circulating concentrations of ox-LDLs that are captured and internalized by scavenger receptors (SRs). Although the key role of SR-dependent ox-LDL signaling in atherogenesis is established, its contribution to the susceptibility of developing cancer has not been addressed yet.

Hirsh and colleagues [173] used the transcriptional expression profiling in two isogenic models of cellular transformation to identify a common transcriptional signature involving inflammatory and metabolic pathways. It linked the cancer gene signature to metabolic diseases that include obesity, diabetes and atherosclerosis. *OLR1* is among the genes that make up this signature, which indicates a more significant involvement of metabolism and neoplastic transformation and suggests molecular links between atherosclerosis and cancer. Observed physiological conditions show LOX-1 is necessary to maintain the structural integrity of tissues, although a rise of its activity is associated with cancer cell invasion (Figure 8).

Through a siRNA-mediate inhibition approach, the depletion of *OLR1* suppresses growth of MCF7 (breast cancer), HepG2 (hepatocellular carcinoma) and HeLa (cervical cancer) cells while affecting the growth of non-transformed cell lines. Depletion of *OLR1* has been found to block morphological transformation, inhibit cell motility and reduce tumorigenicity in different cancer cell lines. As such, *OLR1* is important for the cellular transformation and maintenance of the transformed state as confirmed by the subsequent inhibition of *OLR1* in the MCF10A ER-Src model which reduced NF-κB and inflammatory and hypoxia pathways, thus elucidating the strong connection between cellular transformation and atherosclerosis [173].

The expression of *OLR1* mRNA is modulated in different cancer tissues. The meta-analysis of the gene expression profile of about 950 cancer cell lines stored in the Gene Expression Atlas at the EMBL-EBI database (http://www.ebi.ac.uk/gxa/gene/ENSG00000173391#) revealed that *OLR1* is specifically upregulated in 57% of bladder and cervix cancer cells, 11% of mammary gland cancer cells, 10% of lung cancer cells and in 20% of colorectal cancer (CRC) cells. Moreover, recent studies have shown that high LOX-1 expression was a significant prognostic factor in various cancers, such as advanced-stage prostate cancer [175], colorectal cancer [176] and squamous non-small cell lung cancer [177]. Consistent with these findings, it was reported that upregulation of LOX-1 by ox-LDLs leads to tumor angiogenesis and to an increased cell proliferation in prostate cancer cells. This data depicts a positive correlation between obesity factors and the expression of proliferation and pro-angiogenic markers [175].

A microarray analysis on *OLR1* KO mice demonstrated that abrogation of LOX-1 in tandem with inhibition of NF-κB target genes causes a profound inhibition of rate-limiting enzymes involved in lipogenesis. These include fatty acid synthase (FASN) (−5.9-fold), ATP-citrate lyase (−1.7-fold), acetyl-coenzyme A carboxylase α (−1.9-fold), stearoyl-CoA desaturase 1 (−5-fold) and ELOVL family member 6, elongation of long chain fatty acids (−3-fold) which suggest that LOX-1 may have at least two independent mechanisms of pro-oncogenic action through activation of NF-κB signaling pathway and a novel function as a potent regulator of lipogenesis [170]. *OLR1* may acts as an oncogene through the activation of NF-κB target genes regulating expression of metabolic oncogene FASN (OMIM *600212) [178]. LOX-1 potentially plays a very important role in the targeted therapy of 15%–30% of breast tumors that are HER2-enriched, a mediator of the FASN−HER2 axis.

Individuals with high levels of circulating oxidized low-density lipoproteins and atherosclerotic plaques are more prone to develop colorectal cancer. CRC tissue produces and accumulates an excess of ox-LDLs, which suggests a correlation between lipid dysfunction and malignant transformation. The significance of LOX-1 is further supported by the observation that statins protect against carcinogenesis by inhibiting cholesterol production [179] and, among patients with newly diagnosed coronary artery disease, the prevalence of CRC is greater [180].

The peculiar function of LOX-1 as oncogene has been demonstrated in colorectal cancer underlining the connection between metabolism and cancer insurgence. Murdocca and coworkers documented the upregulation of LOX-1 expression in the different stages of human colon tumorigenesis with its relation to the grade and the stage of the disease. To further demonstrate the role of LOX-1 in colon cancer, they downregulated expression with siRNA in vitro and evaluated influences of LOX-1 levels on the neoplastic phenotype of colorectal cancer cell lines, DLD-1 and HCT8. The in vitro knockdown of LOX-1 in both cell lines impaired proliferation rate and the maintenance of cell growth and tumorigenicity [176].

Downregulation of LOX-1 expression in DLD-1 cells influenced butyrate presence and consequently results in a marked increase of the histone H4 acetylation pattern. The shifting of acetylation pattern suggests a possible involvement of LOX-1 in epigenetic modulation of transcription of tumor suppressor genes [176].

These pathological conditions of cell transformation are associated with an increased oxidative damage to lipids, proteins and DNA [181]. However, few epidemiologic studies have investigated the relationships between lipid peroxidation and colorectal cancer [182,183,184].

Furthermore, LOX-1 expression being correlated to the grade and stage of a tumor may act as a molecular link among metabolism, inflammation and cancer, in addition to its potential as biomarker and molecular therapeutic target.

## 10. The Potential of Targeting LOX-1 in Cardiovascular Disease and Tumors

Acute myocardial infarction (AMI), the most dramatic consequence of atherosclerosis, has been the major cause of mortality and morbidity among late-onset diseases in many industrialized countries with a Western lifestyle [185]. Since the incidence of cardiovascular pathologies and the aging of the population are increasing, the development of new prevention programs is crucial for population wellness and economics [186].

Given the relevant role of ox-LDLs in atherogenesis, a prevention approach is undoubtedly aimed to reduce the plasma ox-LDL levels by using different compounds such as naturally occurring antioxidants and/or antihypertensive agents. These agents either inhibit ox-LDL formation or remove ox-LDLs from circulation, thus preventing atherogenesis [187].

**Figure 9:**
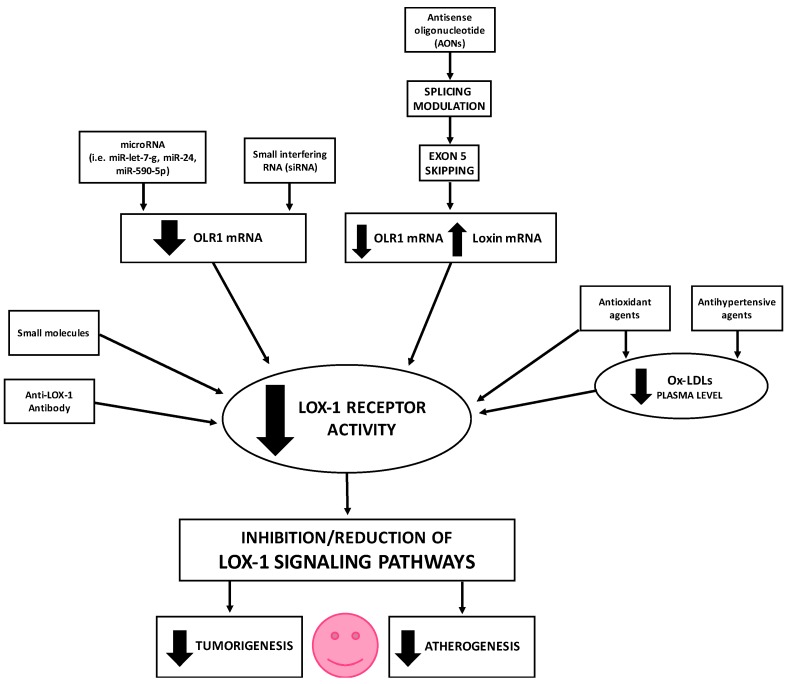
Schematic representation of the therapeutic approaches aimed to lower LOX-1 expression and inhibit/reduce LOX-1 signaling pathways. **Thick arrows** indicate up-regulation/activation (**up arrows**) or down-regulation/inhibition (**down arrows**).

Many therapeutic approaches aimed to lower LOX-1 expression and activity, thus inhibiting or reducing LOX-1 signaling pathways, have been studied and are still object of research (Figure 9).

Antioxidants, such as tanshionone IIA [188], curcumin [189], berberine [190], and resveratrol [191], also inhibit LOX-1 expression and LOX-1 signaling pathway as a result of reduced-circulating ox-LDLs. Among antihypertensive agents such as calcium channel blockers (CCB), Nifedine has an inhibiting effect on LOX-1. In fact, it prevents the apoptosis of endothelial cells by LOX-1 downregulation [192].

LOX-1 is one of the major mediators of the genesis of atherosclerosis and its expression is regulated by pro- and anti-inflammatory cytokines.

Indeed, LOX-1 deletion reduces atherogenesis in mice subject to high-cholesterol diets [193] and protects against plaque instability in conditions of hypercholesterolemia [194].

To this purpose, an interesting therapeutic approach could point to inhibit or reduce LOX-1 overexpression.

Different studies in the literature report the use of anti-LOX-1 antibodies for clinical and therapeutic aims in the cardiovascular field. In vivo studies on myocardial ischemia-reperfusion injury rat model show an increase of myocardial metalloprotease expression and leucocyte recruitment in the control group while the administration of LOX-1 antibody led to a reduction of these effects and of the myocardial infarct area size [195,196]. In addition, the incidence of cardiac hypertrophy in ApoE-KO transgenic mice subjected to a fat- and cholesterol-rich diet was also significantly nullified by anti-LOX-1 antibody treatment [197]. Furthermore, the treatment also demonstrated reduction in lipid deposits in the mesenteric arteries of stroke-prone spontaneous hypertensive rats [198] and protection from pro-atherogenic effects of air pollutants [199].

LOX-1 is also valuable for molecular imaging as demonstrated by Ishino and colleagues [200] who used a Technetium-99-labelled anti-LOX-1 antibody for atherosclerosis detection. The validity and functionality of anti-LOX-1 target mechanisms are apparent; however, it can be problematic because the oral administration of protein is not yet practical. In the meantime, the use of anti-LOX-1 probes show potential in risk stratification of cardiovascular disease.

Antisense RNA is undoubtedly an effective therapeutic strategy for monogenic diseases [201], but this approach has also been successfully exploited to treat complex diseases such as cardiac disease [202].

To date, there is only a discrete number of papers that describe an antisense oligonucleotide (AON) approach to lower LOX-1 expression.

Li et al. [203] suppressed LOX-1 both at mRNA and at protein levels, in human coronary artery endothelial cells (HCAECs) by using antisense oligonucleotides to the 5’-coding sequence of the human *OLR1* gene. Ox-LDL-mediated upregulation of LOX-1 was also suppressed by antisense LOX-1. The pre-incubation of HCAECs with antisense LOX-1 for 48 hours caused both ox-LDL-mediated upregulation of monocyte chemoattractant protein-1 (MCP-1) and monocyte adhesion to HCAEC suppression, whereas sense LOX-1 had no effect. In conclusion, antisense LOX-1 reduced ox-LDL-mediated HCAEC injury and totally inhibited ox-LDL-induced MAPK activation.

We used Schizophyllan (SPG), a polysaccharide that belongs to the β(1−3)-glucan family, as a delivery system for antisense *Olr1* oligonucleotides (SPG/Olr1AS) in ApoE knockout mice and found significant downregulation of *Olr1* mRNA and Lox-1 protein in the aorta of mice [204]. 

Using modified antisense oligonucleotides (AONs), endogenous AS has been successfully modulated [205,206,207,208]. Specifically, AONs directed against RNA regions containing splicing-altering SNPs modulated AS. The complementarity of AONs to the splice sites flanking endogenous *OLR1* exon 5 may induce exon 5 skipping and a consequent reduction of ox-LDL uptake in cells in culture [157].

MicroRNA (miRNA) can also be used for an effective therapeutic strategy aimed at lowering LOX-1 expression. MiRNAs are small endogenous noncoding RNAs approximately 22–26 nucleotides in length that regulate mRNA levels of various protein-coding genes [209,210,211,212,213,214].

There are numerous reports of LOX-1 expression regulation by microRNAs in different cell lines such as human smooth muscle cells (SMCs) and human umbilical vein endothelial cells (HUVEC).

A binding site for miRNA let-7g was found in the 3’ UTR of *OLR1* mRNA. Transfection of miRNA let-7g resulted in the inhibition of ox-LDL-induced expression of LOX-1 [215]. Another study reported that the miRNA let-7g blocked LOX-1 expression as well as ox-LDL uptake and proliferation of human aortic smooth muscle cells (HASMCs) [216]. In particular, hsa-let-7g reduces SMC apoptosis through cytochrome c and Smac/Diablo downregulation and Bcl-xL and Bcl-2 upregulation. Moreover, miRNA let-7g is involved in the reduction of ox-LDL-induced increase of NADPH oxidase, p47 expression and consequent intracellular ROS generation, p44/42 mitogen-activated protein kinase (MAPK) phosphorylation and NF-κB p65 expression [216].

Transfection of HUVECs with miR-590-5p mimic and subsequent treatment with ox-LDLs inhibited the ox-LDL-mediated angiogenesis processes as capillary tube formation, cell proliferation and migration and pro-angiogenic signals.

This inhibitory effect is mediated by LOX-1 inhibition at the translational level and confirmed by luciferase assay [217].

A different study demonstrated that miR-590-5p downregulation promotes Ang II-induced endothelial cell apoptosis because of an increase in LOX-1 expression and consequent ROS generation. Thus, restoration of miR-590-5p and the consequent blocking of LOX-1 receptor could be therapeutically exploited to alleviate endothelial cell apoptosis [218].

We identified the presence of a hsa-miR-24 binding site in the 3’ UTR of *OLR1* that is “naturally” mutated by the presence of a single nucleotide polymorphism (SNP), rs1050286 SNP. We demonstrated that rs1050286 SNP significantly affects miR-24 binding affinity to the 3’ UTR of *OLR1* causing a more efficient post-transcriptional gene repression in the presence of the G allele. On this basis, rs1050286 SNP on the 3’ UTR of *OLR1* may contribute to modify LOX-1 susceptibility to AMI and CAD indicating rs1050286 SNP screening could help to stratify patients risk [219]. Accordingly, a very recent case-control on 526 Chinese patients with atherosclerotic cerebral infarction (ACI) and 640 healthy controls showed that the rs1050283 A allele of LOX-1 was strongly associated with an increased risk for ACI and augmented *OLR1* expression at mRNA, protein and its serum-soluble form (sLOX-1) levels [220].

Another potential therapeutic strategy aimed at lowering LOX-1 expression includes small interfering RNA (siRNA). SiRNA can prevent ox-LDL-induced activation of Rho A (Ras homologue gene family, member A), Rac 1 (Ras-related C3 botulinum toxin substrate 1) [221] and partially inhibit CRP (C-reactive protein) binding [222]. Fujita and colleagues observed a downregulation of LOX-1 expression after siLOX-1 transfection in bovine aortic endothelial cells [223].

Other small molecules such as procyanidins and polyphenol compounds present in red wine and apples also prevent binding of ox-LDLs to LOX-1 [224].

Pretreatment with metformin, a widely used oral antihyperglycemic agent for the treatment of type 2 diabetes, reduces the level of oxLDL-induced LOX-1 upregulation involving the SIRT1/AMPKα/PKCα/NADPH oxidase pathway [225]. In addition, the involvement of Atorvastatin in decreasing cell proliferation was recently demonstrated. The mechanism of action of Atorvastatin on cell proliferation is still obscure but could be represented by the inhibition of LOX-1 action by physical interaction interfering with the LOX-1-related NF-κB activation and p21 activity blocking the cell cycle [226].

A deeper knowledge and characterization of LOX-1 isoforms as disease-specific splice variants can elucidate its mechanism and find potential therapeutic targets for cancer and atherosclerosis. In addition, the modification of the splicing pattern should be a promising endeavor. Furthermore, the study of their expression specificity could lead to variant-specific treatments able to target affected cells with minimal impact on healthy tissue, an important obstacle for any therapeutic approaches. The modulation of the alternative splicing represents a very elegant approach to control *OLR1* overexpression and promote the expression of *Loxin*, the protective isoform, instead. However, in our perspective, a direct approach aimed to strongly reduce *OLR1* isoform expression or block the activity of LOX-1 receptor (Figure 9) appears to be a more successful strategy. Indeed, in this way, it would be possible to quickly keep under control the effects of LOX-1 overexpression and abolish, or at least reduce, the activation of downstream pathways involved in many steps of atherogenesis and tumorigenesis (Figure 8).

## 11. Conclusions

Molecular therapeutic approaches, such as antibodies, antisense oligonucleotides, siRNA, and miRNA, are fast-emerging tools of biotechnology that may inhibit LOX-1 and its splice isoforms laying a foundation for the development of therapeutic strategies against atherosclerosis and tumors linked to LOX-1 overexpression.

Therefore, the regulation of *OLR1* splicing and the expression pattern of *OLR1* splice isoforms in tumors and atherosclerotic lesions deserve further investigation. Disease-associated splice isoforms represent not only potential diagnostic biomarkers but also novel and effective therapeutic targets for the pathologies associated with *OLR1* overexpression.

## Figures and Tables

**Table 1:** Location of single nucleotide polymorphisms (SNPs) in the linkage disequilibrium (LD) block in *OLR1* genomic sequences. Numbers represent genomic coordinates in the human genome (GRCh38/hg38 of NCBI genome browser dbSNP 149).

*OLR1*	SNP	Haplotype Risk	Haplotype Non-RISK	Genomic Position
Intron 4	rs3736232	C	G	chr12:10160759
	rs3736234	T	C	chr12:10160535
	rs3736235	G	A	chr12:10160476
Intron 5	rs17174597	G	A	chr12:10160092
	rs13306593	T	G	chr12:10160049
3’ UTR	rs1050283	T	C	chr12:10159690
